# *In Vitro* Cercaricidal Activity, Acute Toxicity, and GC/MS Analysis of Some Selected Ghanaian Medicinal Plants

**DOI:** 10.1155/2023/4589424

**Published:** 2023-09-15

**Authors:** Bright Osei-Mensah, Yaw Duah Boakye, William Kofi Anyan, Theresa Appiah Agana, Eugene Agyei Aboagye, Ivy Bentil, Elvis Suatey Lomotey, Francis Adu, Christian Agyare

**Affiliations:** ^1^Department of Pharmaceutics, Faculty of Pharmacy and Pharmaceutical Sciences, Kwame Nkrumah University of Science and Technology, Kumasi, Ghana; ^2^Department of Parasitology, Noguchi Memorial Institute for Medical Research, University of Ghana, Legon, Ghana; ^3^Department of Pathology, Manhyia District Hospital, Kumasi, Ghana

## Abstract

Schistosomiasis is a human parasitic disease caused by the *Schistosoma* species and is recognised in public health as second to malaria in terms of its socioeconomic impact on humans. Four local plants native to many tribes in Ghana and known for their medicinal properties against some diseases were assessed for their cercaricidal activity against *Schistosoma mansoni* cercariae. The plants, namely, *Newbouldia laevis* stem bark (NLSB), *Spathodea campanulata* stem bark (SCSB), *Momordica charantia* leaves (MCL), and *Ocimum viride* leaves (OVL), were extracted for their active metabolites using methanol. Preliminary phytochemical screening was carried out on all plant extracts and powdered samples. The crude extracts were tested against *S. mansoni* cercariae *in vitro* using *Balanites aegyptiaca* as the positive control. The percentage of mortalities for each extract was recorded. Gas chromatography/mass spectrometry (GC/MS) analysis was conducted on all the plant extracts. Phytochemical analysis revealed the presence of saponins, glycosides, triterpenoids, sterols, alkaloids, flavonoids, and tannins in almost all the extracts. GC/MS analysis showed the presence of medicinally important active volatile compounds in each extract such as thymol, n-hexadecanoic acid, phytol, and maltol. All four plants showed relatively different levels of activity against *S. mansoni* cercariae at different times and concentrations. The LC_50_ values of the plant extracts were determined at the end of the assay. At 240 min, NLSB, SCSB, MCL, and OVL extracts had LC_50_ values of 487.564, 429.898, 197.696, and 0.129 *μ*g/mL, respectively. Hence, this study revealed the potency of *Ocimum viride* leaves, *Momordica charantia* leaves, *Spathodea campanulata* stem bark, and *Newbouldia laevis* stem bark against *S. mansoni*. These plants could therefore be exploited as possible candidates for curbing schistosomiasis.

## 1. Introduction

As established by the Centre for Disease Control (CDC), schistosomiasis, also known as bilharzia, is a parasitic disease caused by a trematode (blood fluke) parasite of the genus *Schistosoma* [1]. Schistosomiasis remains a major infectious disease and is recognised in public health as second to malaria in terms of socioeconomic impact on humanity [2]. However, schistosomiasis is classified as a neglected tropical disease (NTD) [3]. There are several species of *Schistosoma* (S), but the five common human species are *S. haematobium*, *S. mansoni*, *S. japonicum*, *S. mekongi*, and *S. intercalatum*. The first three are considered the major human species that affect humans, even though the remaining two (*S. mekongi* and *S. intercalatum)* may also cause systemic human disease. Unlike other trematodes, which are hermaphroditic, *Schistosoma* spp. are dioecious [1].

The prevalence of schistosomiasis in both tropical and subtropical areas of Africa, especially among poor communities that lack access to safe drinking water and hygienic conditions, is said to be very high. In 2019, prophylactic treatment of people suffering from schistosomiasis was estimated by the WHO to be around 236.6 million globally with Africans constituting about 90% of the numbers. Sub-Saharan Africa alone is known to record an alarming number of about 192 million cases with a prevalence rate higher in Nigeria (29 million), Tanzania (19 million), Ghana, and Congo (15 million) [4]. In Ghana, the estimated nationwide prevalence as reported by Kulinkina et al. stands at 23.3%, while the localized prevalence level is above 50% [5]. Also, per the Ghanaian District Health Information Management System (DHIMS), a total of approximately 25,000 cases of schistosomiasis were reported in 2010.

Chemotherapy with praziquantel (PZQ) remains the mainstay drug for morbidity control [6]. Although PZQ is reported to have a cure rate of 60 to 90% in both adults and children at a single dose of 40 mg/kg, it is selectively more effective against adult schistosome worms, while its activity against immature worms is abysmal. Hence, this accounts for the readministration of PZQ to an infected person to get rid of the young schistosomes that had since developed into adult worms after the first dose [7]. However, one very potent plant against the early stages of schistosomes (cercariae) had been reported in the stem bark of *Balanites aegyptiaca* even at lower concentrations of 1000 ppm. The stem bark of *Balanites aegyptiaca* as an ointment showed promising results in preventing the penetration of cercariae into human skin and is yet to go through preclinical and clinical trials [8]. Aqueous extract of the seed of *Balanites aegyptiaca* was treated against *S. mansioni* cercariae and showed reasonable efficacy. Different parts of *Balanites aegyptiaca* such as the fruit, mesocarp, and endocarp also exhibited good molluscicidal potency against snail vectors, *Biomphalaria pfeifferi* and *Lymnaea natalensis* [9].

Since the treatment of schistosomiasis has almost always been fully dependent on praziquantel, a potential threat of selective resistance by *Schistosoma* is highly possible and expected [10]. The issue of PZQ being the only drug of choice used against schistosomiasis to treat millions of people for the past 40 years has always been a concern. For example, Egypt, known to have resorted to the use of PZQ against schistosomiasis for over 10 years, has reported low cure rates in response to PZQ by some strains of *Schistosoma*, though some scientists share contrary views on the possible existence of resistance [11]. To back this claim of possible resistance to PZQ by *Schistosoma*, a study conducted in Kisumu, Kenya, had isolates of *S. mansoni* showing lower susceptibility to PZQ [12]. It is therefore imperative to find an alternative therapy to complement PZQ usage.

The onset of *Schistosoma i*nfection in the definitive host occurs when humans get into close contact with freshwater in which infected people have urinated or defecated to release ova that hatch to release a larval form known as miracidium. Miracidium then finds a suitable intermediate host (snail) to penetrate and undergoes two sporocyst generations that migrate to the hepatic and gonadal tissues [2]. After 2 to 4 weeks, the sporocyst develops to form the next larval form known as cercariae (infective form) which finds a human host and burrows into the skin, discarding its forked tail to form schistosomulae [13]. Treatment against any of the aforementioned stages of the parasite's life cycle could be a possible breakthrough.

Natural products with schistosomicidal activity have mainly been identified from plant sources [7]. The main metabolites of plants (phenolic compounds, terpenes, alkaloids, and peptides) continue to be a reliable approach to the development of novel drugs for the treatment of human diseases, particularly in developing countries [14]. These natural products have become coveted worldwide due to their perceived lesser side effects and low toxicity profile compared to synthetic drugs [15]. In Africa, a host of plants including *Abrus precatorius*, *Pterocarpus angolensis*, and *Antidesma venosum* have been identified via plant screening to have anthelminthic potential, but their cercaricidal activity has not been verified empirically [16]. Across West Africa, the natives use *M. charantia* against worms (Lumbricus) and some parasitic infections [17]. The leaves of *M. charantia* are reported to be used in the treatment of malaria, menstrual troubles, constipation, hepatitis, and helminthiases [18]. *N. laevis* is used widely in Africa to cure many diseases which include malaria and fever, stomach ache, sexually transmitted diseases (STDs), and breast cancer [19]. In Ghana, the stem bark of *S. campanulata* is used traditionally to treat dysentery and diarrhoea [17]. As indicated by Bhagat et al. [20], *Ocimum* leaves are used traditionally as a decoction for fever and cough and extensively in treating rheumatism and lumbago. In conjunctivitis, the fresh juice from the leaves is used as eye drops and also as a remedy in situations of excessive discharge of mucus in the nose or throat. This study therefore sought to assess the safety, possible chemical compositions, and cercaricidal activity of *Momordica charantia*, *Newbouldia laevis*, *Ocimum viride*, and *Spathodea campanulata* against *S. mansoni* cercariae.

## 2. Materials and Methods

### 2.1. Collection, Preparation, and Extraction of Crude Plant Extracts

The stem barks of *Newbouldia laevis* (voucher number KNUST/HM1/2021/SB010) and *Spathodea campanulata* (voucher number KNUST/HM1/2019/SB008) and the leaves of *Ocimum viride* (voucher number KNUST/HM1/2021/L003) and *Momordica charantia* (voucher number KNUST/HM1/2021/L002) were collected in the month of October from Sunyani, Chiraa, and Fiapre in Bono Region, Ghana, in 2020 and authenticated by Dr. G. H. Sam, a botanist at the Department of Herbal Medicine, Faculty of Pharmacy and Pharmaceutical Sciences, Kwame Nkrumah University of Science and Technology, Kumasi, Ghana (Table 1). All these plant specimens were collected based on their antimicrobial properties that were reported in some studies and also due to their abundance and patronage by local folks in the Ghanaian communities. The collected plant parts were cleaned of all foreign matter and dried at room temperature (25°C) for 2 weeks. With slight modifications from previous studies [21], the plant materials after being air-dried were reduced to smaller coarse particles by milling. Precisely 100 g of each pulverized plant material was soaked in absolute methanol (cold maceration) in glass containers at 25°C with frequent agitation for 3 days. Afterwards, the mixture was filtered using Whatman Number 1 filter paper, and the resulting filtrate was concentrated via evaporation at 40°C under reduced pressure using a rotary evaporator. Finally, the recovered filtrates were oven-dried at 45°C. The extracts were then weighed and stored in labelled airtight glass containers at 4°C in the refrigerator until needed.

### 2.2. Collection and Maintenance of Snail Vectors

Snails (*Biomphalaria* species) suspected to be infected with *S. mansoni* cercariae were collected from their natural habitats at Tomefa along the Densu River in the Ga South Municipality, with geographical coordinates of N 05.57379° and W 000.37714° in the Domeabra-Obom constituency in Accra, Ghana, and transported to the snail laboratory of the parasitology department at the Noguchi Memorial Institute for Medical Research (NMIMR) in beakers filled with distilled water. At the laboratory, the snails were fed with fresh lettuce and kept in the dark until needed to prevent shedding.

#### 2.2.1. Harvesting of Cercariae from Snail Vectors and Preparation of Cercariae Suspension

Using the method described by Milligan and Jolly with slight modifications [22], all the field-collected snails were once again washed thoroughly with clean dechlorinated water about three times to get rid of almost all debris before exposure. With the aid of forceps, snails were picked individually into separate wells of a 24-well culture plate (1 in each well) containing 1 mL of distilled water. The culture plate was then covered with its lid and positioned under an artificial light about 9 to 12 inches away for a maximum of 30 minutes to induce the shedding of cercariae from infected snails. The temperature of the room was set to 28°C ± 1 before the exposure. After 30 min of exposure, all the snail hosts were carefully filtered out of the wells. The wells were then observed keenly under an inverted microscope to ascertain the presence of human cercariae. All the wells that had cercariae in them were carefully pulled together into a beaker with the aid of a Pasteur pipette. An estimation of the number of cercariae present in the total volume of cercariae suspension pulled together was made per every 50 *μ*L of suspension pipetted and counted under the inverted microscope with the help of a tally counter. The 50 *μ*L count was repeated four (4) times, and the averages were taken for the estimation.

#### 2.2.2. *In Vitro* Cercaricidal Activity of Crude Plant Extracts

A two-fold serial dilution (1000, 500, 250, 125, 62.5, and 31.25 *μ*g/mL) was made for each of the test plant extracts from their stock concentrations of 200 mg/mL using distilled water as the diluent. Precisely 500 *μ*L of each extract concentration was transferred in triplicates into a 24-well culture plate already containing 500 *μ*L of cercariae suspension from the lowest to the highest concentration. The wells were then observed under an inverted microscope at specific time intervals (15, 30, 60, 90, 120, 150, 180, and 240 min) at 28°C to count the number of dead cercariae in each well for the various test concentrations. Cercariae were considered dead with the aid of an inverted microscope (×40 magnification) when they became immobile even after agitation, had a sunken body, and/or had a detached head from the body [23]. *Balanites aegyptiaca* was used as the positive control, and distilled water and cercariae mixture were used as the negative control. The experiment was conducted in triplicate.

### 2.3. Phytochemical Screening

Preliminary qualitative screening of both the prepared crude extracts and the powdered plant samples was carried out to ascertain the presence/absence of alkaloids, flavonoids, glycosides, triterpenoids, sterols, saponins, and tannins.

#### 2.3.1. Tannin Test

Fifty milligrams of the extracts and powdered samples were each put in a test tube containing 10 mL of distilled water, boiled for 5 min, stirred, and filtered. A 0.1% *v*/*v* ferric chloride solution was then added in a few drops to 2 mL of the filtrate. The presence of a brownish-green colour justified the presence of tannins [24].

#### 2.3.2. Flavonoids Test

The crude extract and powdered sample of weight 50 mg were each dissolved in distilled water and filtered. A piece of white paper was passed through the filtrate and made to dry. The dried paper piece was exposed to a 10% *v*/*v* ammonia solution. An intense yellow colouration was considered evidence of the presence of flavonoids [25].

#### 2.3.3. Saponin Test

The prepared extract and powdered sample were each diluted with distilled water and agitated vigorously at room temperature in test tubes for 15 minutes. The persistent formation of a 1 cm-thick layer of foam indicated the presence of saponins [24].

#### 2.3.4. Test for Glycosides

To twenty milligrams of the extract and powdered plant sample each, 1 mL of Fehling's A and Fehling's B solutions were added and boiled in a water bath for 5 minutes at 100°C. The formation of an initial yellow colour and then a brick-red precipitate was considered evidence of the presence of glycosides [25].

#### 2.3.5. Test for Alkaloids

An amount of 0.5 g of plant material (extract or powdered sample) was dissolved in dilute hydrochloric acid, heated for 20 min, cooled, and filtered. One milligram of each filtrate was treated with a diluted potassium mercuric iodide solution for 2 min (Mayer's test). A yellow colour precipitate formation indicated the presence of alkaloids [25].

#### 2.3.6. Triterpenoids Test

An amount of 5 mg of the plant material (extract or powdered sample) was put in 2 mL of chloroform, and 1 mL of concentrated acetic anhydride was added. To the solution in a test tube, 1 mL of concentrated H_2_SO_4_ was meticulously added down the side. Finally, the formation of a cherry-red colour at the chloroform-sulphuric acid interface showed the presence of triterpenoids [26].

#### 2.3.7. Test for Sterols

Using Salkowski's test, 5 mL of chloroform was added to 2 mL of plant material (crude extract or powdered sample) and filtered using filter paper. One millilitre of concentrated H_2_S0_4_ was added along the walls of the test tube to 2 mL of the chloroformic filtrate. A colour formation of reddish brown at the lower layer was considered evidence for the presence of sterols in the test samples [27].

### 2.4. Gas Chromatography-Mass Spectroscopy (GC/MS) Analysis

GC-MS analysis of the methanol extracts of *M. charantia* leaves, *O. viride* leaves, *S. campanulata* stem bark, and *N. laevis* stem bark was carried out using the Perkin Elmer GC Clarius 500 analyzer interfaced with the Perkin Elmer Mass Spectrometer (Clarius SQ 8 S). Data from samples were obtained on an Elite-1 (100% dimethyl polysiloxane) coupled with the capillary column (30.0 × 250 *μ*m). With helium (99.999%) as the carrier gas at a flow rate of 1.0 mL/min, a 1 *μ*L aliquot of sample was injected into the column with the temperature of the injector at 250°C and that of the detector (Turbo-Mass) at 280°C. The temperature programme (oven temperature) began at 100°C, held for 2 minutes, and increased steadily to 280°C (5°C/min). Extracts were dissolved in methanol and fully ran for 40 minutes. The compounds in each extract were obtained in the form of a mass spectrum by electron ionization at 70 eV with a scanning interval of 1 second and fragments maintained from 50 to 500 Da. From the database of the National Institute Standard and Technology (NIST) with over 62,000 patterns of mass spectra of diverse compounds, the interpretation of data from GC-MS analysis on all extract samples was conducted.

### 2.5. Acute Oral Toxicity Study of Plant Specimen per OECD Guideline

#### 2.5.1. Test Animals

Female Sprague-Dawley rats of weight ranging from 152.4 to 251.9 g were categorized into five (5) groups in well-moulded stainless steel cages with each group having a total of three (3) rats. These rats were obtained from the animal house, Department of Pharmacology, Kwame Nkrumah University of Science and Technology, Kumasi, Ghana. After the acquisition, rats were allowed to adapt to new conditions before use for a period of two weeks, during which food (rat feed) and water were made available to them. Per the National Institute of Health guidelines, rats were properly handled with care. They were kept under a 12/12 h light/dark cycle and an ambient temperature of 25°C. Approval was obtained from the Animal Ethical Committee, Faculty of Pharmacy and Pharmaceutical Sciences, KNUST, Kumasi, Ghana, to carry out all animal studies.

#### 2.5.2. Acute Toxicity Study on Oral Administration of Plant Extracts (Limit Test)

Female Sprague-Dawley rats were put into four (4) treatment groups and an untreated group. The rats were preweighed, and the dose was calculated based on individual weights before treatment. All three (3) rats in each treatment group were administered one of the test plant materials at a single dose of 2000 mg/kg body weight since the plant species under study are publicly coveted in terms of their usage and no toxic effects have yet been identified. The route of administration was oral, and the whole method employed was per OECD Guideline 423 (Organization for Economic Cooperation and Development). The untreated group was administered orally with distilled water only. The group of three (3) rats was organized as follows:

Group 1: Methanol extract of the leaves of *M. charantia*

Group 2: Methanol extract of the stem bark of *S. campanulata*

Group 3: Methanol extract of the leaves of *O. viride*

Group 4: Methanol extract of the stem bark of *N. laevis*

Group 5: Untreated group administered with distilled water

#### 2.5.3. Postadministration of Plant Extracts

After oral administration of extracts, the rats were closely observed and monitored intermittently every one (1) hour for four (4) hours on the very first day and once daily for 14 days. Changes such as behavioural, skin and eye colour, body weight (weekly), constipation, urination, tremor, convulsions, and death (major endpoint) were monitored [21].

#### 2.5.4. Removal of Vital Organs

On the 14^th^ day of the acute-toxicity studies, rats were humanely sacrificed, and protocol-specified organs (liver and kidney) were surgically removed. Macroscopic examination of the said organs was carried out, and they were weighed using an analytical balance. The relative organ weight (ROW) of each rat was hence calculated as a ratio of the organ weight to the whole body weight on the day of sacrifice expressed as a percentage [28]. Tissue fragments of the kidney and liver were fixed in 10% neutral buffered formalin, embedded in paraffin wax, sectioned into small pieces of about 4 to 5 *μ*m, and then finally stained using haematoxylin and eosin dyes under standardized pathologic techniques. Histopathological analysis of the various sections was done microscopically under ×40 magnification to observe for any possible signs of toxicity such as leukocyte infiltration, tissue integrity, and injuries (apoptosis, necrosis, and degeneration).

### 2.6. Data Analysis

Data entry management and preliminary summaries from triplicate experiments such as averages, percentages, and graph works were done using Microsoft Excel and Microsoft Word 2013 software. Data from the cercaricidal activity of the plant extracts were subjected to Finney's probit regression analysis using Microsoft Excel 2013 to ascertain the median lethal concentration (LC_50_). A probability value of *p* < 0.05 was considered statistically significant.

## 3. Results

### 3.1. *In Vitro* Cercaricidal Activity

**Figure 1:**
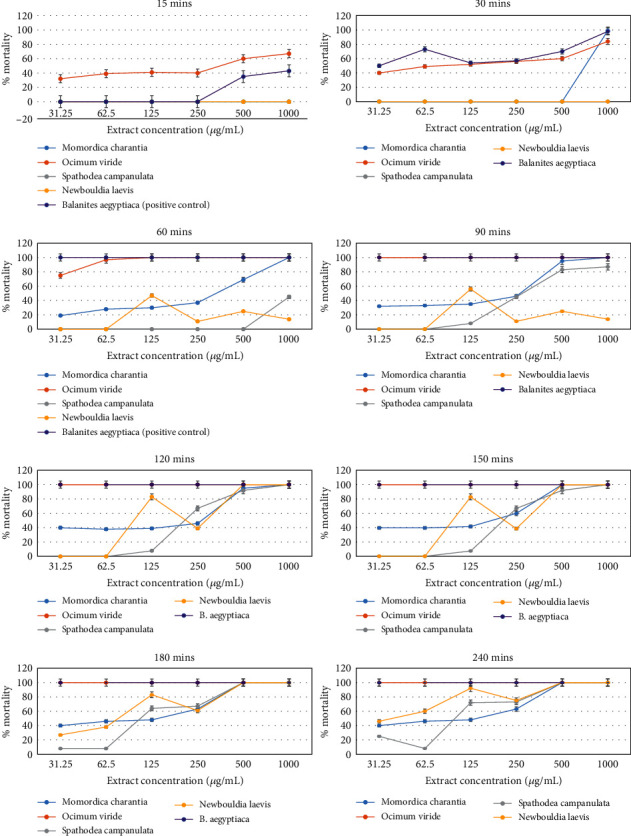
Effect of plant extracts in the time-course mortality rate of *S. mansoni* cercariae.

**Figure 2:**
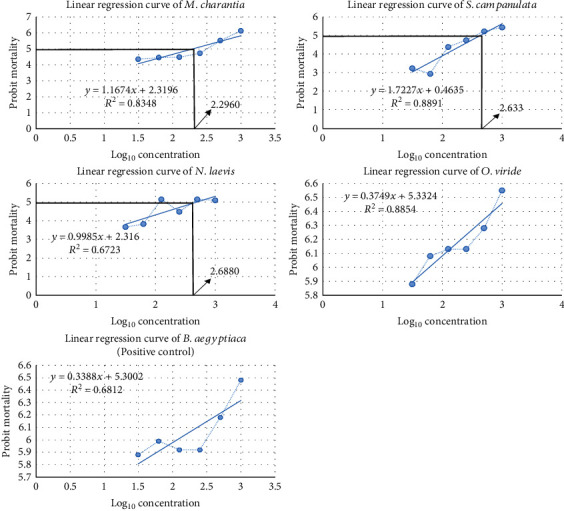
Linear regression curves of plant extracts.

At the first 15 min of exposure, *Ocimum viride* happened to be the only crude extract to show activity from the least concentration to the highest (31.25 to 1000 *μ*g/mL), while *Balanites aegyptiaca* (positive control) showed no killing effect for concentrations ranging from 31.25 to 250 *μ*g/mL. However, *Balanites aegyptiaca* recorded mortalities at concentrations of 500 to 1000 *μg*/mL. The other extracts (*Newbouldia laevis* and *Spathodea campanulata*) showed no mortality until 60 min before a gradual rise was recorded in percent mortality except for *Momordica charantia* which recorded percent mortalities at 30 min for concentrations of 500 to 1000 *μ*g/mL (Figure 1). The rate of kill was higher in *Balanites aegyptiaca*, as 100% mortality was achieved at 60 min for all concentrations, while *Ocimum viride* achieved 100% mortality for all concentrations at 90 min. In contrast to *Balanites aegyptiaca* (positive control), *Ocimum viride*, *Newbouldia laevis*, and *Momordica charantia* recorded 100% mortality in the 150^th^ min only at concentrations ranging from 500 to 1000 *μ*g/mL. *Spathodea campanulata*, however, was the last to record 100% mortality at 180^th^ min only at concentrations ranging from 500 to 1000 *μ*g/mL (Figure 1). The highest average percentage mortality after 240 minutes was against *O. viridae* at 1000 *μ*g/mL, while the lowest percentage mortality was against *S. campanulata* at 31.25 *μ*g/mL (Table 2). *Balanites aegyptiaca* and *Ocimum viride* recorded the least LC_50_ values at the end of 240 min followed by *Momordica charantia*, *Spathodea campanulata*, and *Newbouldia laevis* accordingly with no mortality recorded in the control group (Table 2). The LC_50_ values of *S. mansoni* cercariae against the plant extracts were calculated using probit regression analysis, and all were statistically significant (*p* < 0.05) (Figure 2 and Table 3).

### 3.2. Gas Chromatography-Mass Spectrometry Analysis

GC/MS analysis was performed to ascertain the presence of active volatile compounds that are present in the plant extracts as they may contribute to their medicinal properties. These compounds are enlisted together with their retention times, molecular formula, and peak area.

The chromatogram of the methanol extract of *M. charantia* leaves (MCL) revealed five (5) prominent peaks with n-hexadecanoic acid (C_16_H_32_O_2_) having a larger peak area of 20.50% at a retention time (RT) of 14.128. The remaining peaks of MCL showed the presence of phytol (C_20_H_40_O), 3,7,11,15-tetramethyl-2-hexadecen-1-ol (C_20_H_40_O), thymol (C_10_H_14_O), and 1-chloromethyl-1-(2-propenyloxy)-1-silacyclohexane (C_7_H_13_Cl) at retention times of 16.071 (9.356%), 12.64 (2.681%), 6.152 (1.71%), and 3.20 (1.505%), respectively (Table 4). *S. campanulata* stem bark (SCSB) showed four (4) prominent peaks; Octadecenoic acid, (2-phenyl-1, 3-dioxolan-4-yl) methyl ester, cis-(C_28_H_44_O_4_), n-hexadecanoic acid (C_16_H_32_O_2_), octadecanoic acid (C_18_H_36_O_2_), and hexadecanoic acid, 1-(hydroxymethyl)-1, 2- (C_35_H_68_O_5_) at respective retention times and peak areas of 9.911 (6.444%), 13.78 (6.350%), 16.40 (2.647%), and 11.45 (2.436%) (Table 5). The chromatogram of *N. laevis* stem bark (NLSB) also showed four prominent peaks, with maltol (C_6_H_6_O_3_) having the largest peak area of 9.643% at RT of 3.38, followed by 2-(3-bromo-4-methoxy-4-methylcyclohexyl) propionitrile (C_11_H_18_BrNO) at RT of 3.11 and a peak area of 8.456%. The remaining peaks of NLSB with least peak areas of 2.259% and 2.063% represent 4-((1E)-3-hydroxy-1-propenyl)-2-methoxyphenol (C_10_H_12_O_3_) and benzoic acid, 4-hydroxy-3,5-dimethoxy- (C_9_H_10_O_5_) at retention times of 11.45 and 12.28, respectively (Table 6). *O. viride* leaves (OVL) showed two (2) prominent peaks corresponding to thymol (C_10_H_14_O) and phytol (C_20_H_40_O) compounds occurring at respective RT of 5.95 and 15.78 with peak areas of 6.571% and 2.805%, respectively (Table 7).

### 3.3. Acute Oral Toxicity

**Figure 3:**
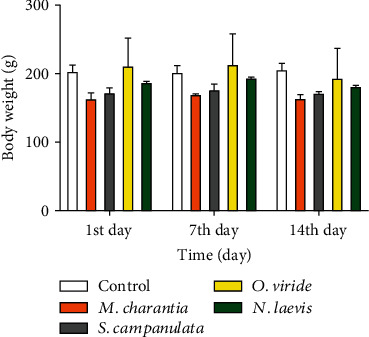
Graphical representation of the changes in body weights of female Sprague-Dawley rats. The control rats saw a slight steady increment from the 1^st^ day through to the 14^th^ day. The treatment groups, *M. charantia*, *S. campaulata*, *O. viride*, and *N. laevis*, however, saw slight decrements on the 14^th^ day.

Assessment of the possible toxic effects of the methanol extract of *S. campanulata* (stem bark), *N. laevis* (stem bark), *M. charantia* (leaves), and *O. viride* (leaves) given as a single dose was based on parameters spelt out in OECD Guideline 423 (acute toxic class method) among which include mortality, body weight, lethargy, coma, convulsions, diarrhoea, tremor, salivation, and any other spontaneous alterations in the general well-being of the rat [21]. From all indications, none of the rats in the treatment groups showed any signs of poisoning compared to the control group. The table below indicates the various observations made after administering the individual plant extracts (Figure 3 and Table 8).

#### 3.3.1. Liver and Kidney Weights

The organ weights of both treatment and control groups were taken as they may show significant differences even if there are no noticeable morphological changes and happen to be a very sensitive indicator of toxicity [29]. The absolute and relative organ weights of the rats showed no significant differences (*p* > 0.05) between the control and treatment groups (Table 9).

#### 3.3.2. Histological Tissue Pathology of Liver and Kidney

**Figure 4:**
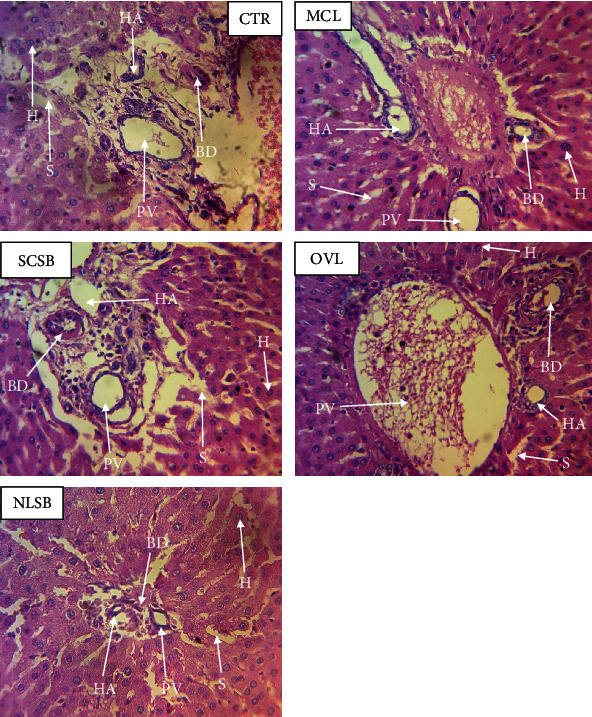
Liver histology, ×400 magnification. The effects of individual plant extracts administered to four different treatment groups of rats (MCL, SCSB, OVL, and NLSB denoting groups 1, 2, 3, and 4, respectively) were observed for any signs of toxicity around the portal triad (BD: bile duct; HA: hepatic artery; PV: portal vein) in both the treated and control (CTR) groups. The control group however was administered with just distilled water. H: hepatocytes around this area and the S: sinusoids were closely observed for any signs of necrosis.

**Figure 5:**
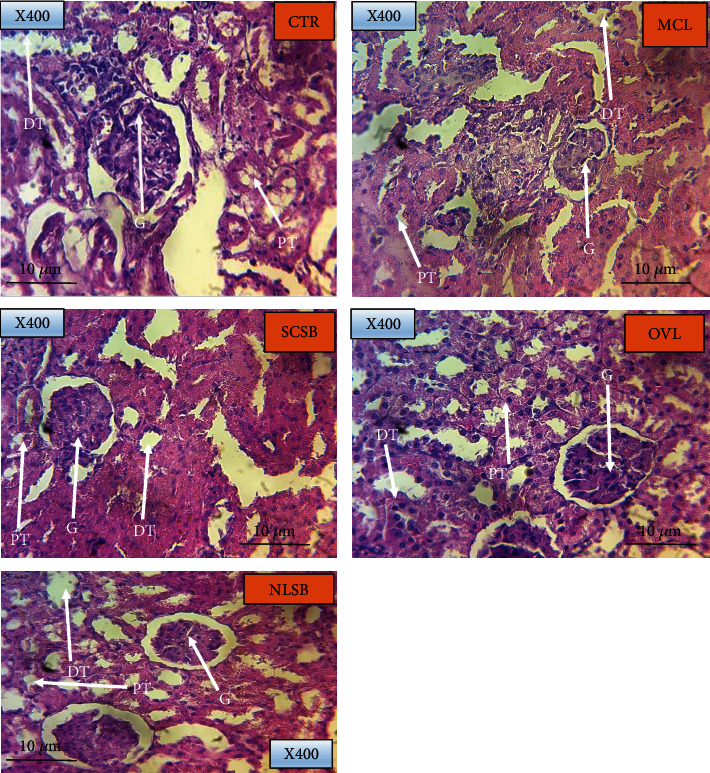
Renal histology, ×400 magnification, scale bar of 10 *μ*m. The effects of individual plant extracts administered to four different treatment groups of rats (MCL, SCSB, OVL, and NLSB denoting group 1, 2, 3, and 4, respectively) were observed for any signs of tubular necrosis at the PT: proximal convoluted tubule and DT: distal convoluted tubule in both treated and control (CTR) groups since the G: glomerulus takes a longer period to damage. The control group however was administered with just distilled water.

A microscopic examination of the histological sections of both the liver and kidney was done to evaluate the architecture of these tissues after administering the various extracts to the control group. The hepatocytes of the liver separated by sinusoids in the treated rats were normal and identifiable as in the control group with no signs of cell necrosis (Figure 4). The portal triad (portal vein, bile duct, and artery) also had their nuclei intact and visible. In the renal parenchyma of treated rats, both tubules (proximal and distal) had a typical appearance as observed in the control group and hence no abnormality. Conclusively, there were no obvious patterns of toxicity in the liver and kidneys of treated rats for all plant extracts (Figures 4 and 5).

## 4. Discussion

The growing impact of schistosomiasis globally has prompted the need for research into medicinal plants as an alternative medicine to resolve this problem. This study sought to look into the curative potentials of some selected Ghanaian medicinal plants against the infective stage of the *Schistosoma mansoni* parasite. The stem barks of *S. campanulata* (SCSB) and *N. laevis* (NLSB) and the leaves of *M. charantia* (MCL) and *O. viride* (OVL) were extracted using the method of maceration in methanol for their phytoconstituents. Though most traditional practitioners rely on water as the medium of extraction for these plants, methanol is used in this study due to its high potency since active plant metabolites dissolve better in organic solvents than water, as reported by Ncube et al. [30].

In this study, the cercaricidal activity of the methanol extract of MCL, OVL, SCSB, and NLSB was ascertained in comparison to *B. aegyptiaca* (stem bark) as a positive control [8]. The extracts showed a linear relationship between the cercariae mortality and individual concentrations (*μ*g/mL) from the probit mortality analysis (Figure 2). All extracts showed significant levels of cercaricidal activity against *S. mansoni* within 4 hours (*p* < 0.05) (Figure 1). The median lethal concentration (LC_50_) of the test samples indicated a stronger cercaricidal activity for OVL as it was at par with the control (Table 3). MCL, SCSB, and NLSB also showed promising activity with a strong and positive correlation between concentrations and probit mortality. This study is in harmony with studies from Damashi et al. [31] and Tekwu et al. [32] which reported the cercaricidal activity of *R. vomitoria* stem bark and cyclosporine, respectively, as dependent on both concentration and time.

The cercaricidal activity of MCL, NLSB, and SCSB can be attributed to the presence of alkaloids since alkaloids isolated from *Teclea nobilis* and *Jatropha elliptica* have been previously reported to have strong cercaricidal activity [33]. The lethality of OVL extract to *S. mansoni* cercariae can also be associated with the presence of triterpenoids since it lacks alkaloids. As indicated by Simoben, triterpenoids from the roots of *Asparagus stipularis* are reported to be potent against schistosomiasis [33]. Also, all flavonoid-containing extracts could have their cercaricidal activity linked to that biomolecule since *Millettia thonningii* is reported to exhibit strong *in vitro* anticercarial activity from flavonoids isolated from it [23].

To identify and authenticate the possible volatile compounds in the various extracts, GC/MS analysis was employed. GC/MS analysis of MCL (Table 4) in this study corroborates with a study by Shehu et al. which reported the presence of n-hexadecanoic acid and phytol [34]. Both compounds have antioxidant and antimicrobial activities [34]. Also, these two compounds were the major chemical constituents among the five compounds present in MCL with area percentages of 20.503% and 9.356% for n-hexadecanoic acid and phytol, respectively. The bioactivity of MCL could therefore be assigned to these two major compounds.

Thymol and phytol were the two prominent compounds found in OVL with respective quantitative area percentages of 6.571% and 2.805% making thymol the major constituent of OVL (Table 7) and hence confirming a study by Bhagat et al. [20]. Thymol is reported to possess antibacterial and antifungal properties [35].

Compounds from SCSB as presented by GC/MS (Table 5) showed four prominent peaks with n-hexadecanoic acid and octadecenoic acid, (2-phenyl-1, 3-dioxolan-4-yl) methyl ester, *cis*- having the highest area percentage (quantity). Wagh and Butle reported that SCSB is devoid of these compounds; however, they indicated that n-hexadecanoic acid was present in the flowers of *S. campanulata* [36]. This could be from how the stem bark was processed even before extraction, and the solvent for extraction could also be a major factor.

Maltol which happens to be the abundant compound in NLSB from GC/MS analysis (Table 6) is known to possess anti-inflammatory properties [37]. Maltol has been reported by Yadav et al. to possess antibacterial activity [38]. Maltol could then possibly be supported by the other three relatively minute compounds present to synergistically confer the antimicrobial activity.

All the detected possible plant compounds conferring on the test plants' cercaricidal activity exhibited similar outcomes of death: immobility and detachment of body parts, and this corroborates with studies by Feussom et al. [23]. The detachment of body parts (head and tail) conforms similarly to the mechanism of action by praziquantel (PZQ) as established by Thétiot-Laurent et al. [39] to induce tetanic contractions in the musculature of adult schistosomes *in vivo* and tegumental damage which begins 5 minutes posttreatment.

Toxicity studies on the extracts were imperative to know the level of safety or limits to which these plants could be lethal or not. In all the treatment groups, there were no mortality, body weight, lethargy, coma, convulsions, diarrhoea, tremor, salivation, and any other spontaneous alterations in the general well-being of the rat (Table 8).

This finding agrees with that of Clarke and Clarke that said substances whose LD_50_ in rats falls below 50-100 mg/kg should be regarded as very toxic, and those with LD_50_ above 500 mg/kg but below 1000 mg/kg are classified as being moderately toxic, while substances whose LD_50_ in rats is above 1000 mg/kg are considered safe or of low toxicity [40].

The body weights of the rats were closely monitored to know their metabolic and physiologic status to prevent the making of any false observations that might be due to abnormalities relating to nutrition and not necessarily the administered extract. There was a slight reduction in the body weights of the treatment groups (*p* < 0.0001). According to Bailey et al. [29] and Shamaki et al. [41], body weight changes can occur through alteration in growth, especially when they contain agents that modify the secretion of growth hormone or somatostatin, or through alteration of hormonal status, e.g., agents that modify the secretion of sex steroids and therefore alter maturational pattern, or through changes in neurotransmitters that affect food consumption, such as agents that affect central serotonergic or dopaminergic systems, reduced palatability of diets containing the experimental compound *opr* through nonspecific systemic toxicity. Therefore, further assessment for signs of toxicity was encouraged to look into their internal organs (liver and kidney). The relative organ weight (ROW) which is another basic indicator of toxicity was analysed but showed no statistically significant changes between the control and treatment groups (*p* > 0.05) (Table 9). Liver and kidney histology showed normal tissue architecture in all treatment groups (MCL, OVL, NLSB, and SCSB) relative to control, affirming no signs of toxicity from any of the extracts administered (Figures 4 and 5). The acute toxicity status of NLSB in this current study is in agreement with Anaduaka et al. [42], who also reported no toxicity.

## 5. Conclusion

The plant extracts used in this study, *Spathodea campanulata* stem bark, *Newbouldia laevis* stem bark, *Momordica charantia* leaves, and *Ocimum viride* leaves, proved effective against *Schistosoma mansoni* cercariae and can be considered potential candidates for novel antischistosomal drug development. This research, however, opens up the idea of topical formulations which could be incorporated into body creams to smear the bodies of village folk, especially children that play in these infected freshwater bodies. That is, applying preventive measures rather than the usual treatment approach to curbing this neglected tropical disease. This will not only reduce cost but also cause the complete extinction of the parasite as a very important stage of its life cycle is truncated. In light of this, selective pressure on the exclusive use of praziquantel will reduce and improve upon its effectiveness as the test plants have prospective potential to complement it. Furthermore, studies on these plants are to be conducted to know the extent of their effectiveness against adult schistosomes now that their potential has been established against the infective cercariae in this study.

This study encourages constructive patronage of these plants by traditional folks as they do not pose any threat to life since these plants showed no signs of toxicity in rats; nonetheless, abusive usage should not be encouraged as further studies on long-term effects were not ascertained.

## Figures and Tables

**Table 1:** Traditional medicinal plants collected for the study.

Plants	Part used	Family	Location	Geographic location	
				Latitude°	Longitude°
*M. charantia*	Leaves	Cucurbitaceae	Fiapre, Bono Region	7.362	-2.346
*S. campanulata*	Stem bark	Bignoniaceae	Sunyani, Bono Region	7.33991	-2.32676
*O. viride*	Leaves	Lamiaceae	Chiraa, Bono Region	7.398	-2.182
*N. laevis*	Stem bark	Bignoniaceae	Sunyani, Bono Region	7.33991	-2.32676

**Table 2:** Average percentage mortalities of plant extracts against *S. mansoni* cercariae.

Extract concentration (*μ*g/mL)	Average % mortality after 240 min ± standard deviation (SD)				
	*Mormodica charantia*	*Spathodea campanulata*	*Ocimum viride*	*Newbouldia laevis*	*Balanites aegyptiaca* (positive control)
1000	87.4 ± 35.31	66.5 ± 45.07	93.9 ± 12.22	53.5 ± 49.99	92.6 ± 20.06
500	69.9 ± 44.32	59.4 ± 49.50	90 ± 18.52	56.3 ± 47.72	88.1 ± 23.90
250	39.4 ± 26.01	39.9 ± 34.02	87 ± 24.45	29.5 ± 28.44	82.13 ± 36.44
125	30.3 ± 19.63	27 ± 33.12	86.6 ± 24.94	55.5 ± 37.48	81.8 ± 36.75
62.5	28.9 ± 18.82	2 ± 3.70	85.6 ± 25.85	12.3 ± 22.05	84.1 ± 35.28
31.25	26.4 ± 17.81	4.1 ± 3.87	80.9 ± 29.09	9.1 ± 16.50	81.3 ± 37.20
Control (no treatment)	0	0	0	0	0

**Table 3:** Summary of log_10_ concentration and corresponding median lethal concentration (LC_50_) of plant extracts determined over the full duration.

Plant specimen	Log_10_ concentration	Antilog concentration (*μ*g/mL) after 240 min (LC_50_)
*M. charantia*	2.2960	197.696 ± 13.579
*S. campanulata*	2.6334	429.898 ± 69.389
*Newbouldia laevis*	2.6880	487.564 ± 69.376
*Ocimum viride*	-0.8866	0.129 ± 0.042
*Balanites aegyptiaca* (control)	-0.8860	0.129 ± 0.051

**Table 4:** Chemical constituents present in the methanol extract of *M. charantia* leaves from GC-MS analysis.

Peak number	Retention time (mins)	Area %	Compound name	Molecular formula	Molecular weight (g/Mol)
1	3.20	1.505	1-Chloromethyl-1-(2-propenyloxy)-1-silacyclohexane	C_7_H_13_Cl	132.63
2	6.152	1.711	Thymol	C_10_H_14_O	150.22
3	12.64	2.681	3,7,11,15-Tetramethyl-2-hexadecen-1-ol	C_20_H_40_O	296.5
4	14.128	20.503	n-Hexadecanoic acid	C_16_H_32_O_2_	256.4
5	16.071	9.356	Phytol	C_20_H_40_O	128.17

**Table 5:** Chemical constituents present in the methanol extract of *S. campanulata* stem bark from GC-MS analysis.

Peak number	Retention time (mins)	Area %	Compound name	Molecular formula	Molecular weight (g/Mol)
1	9.911	6.444	Octadecenoic acid, (2-phenyl-1,3-dioxolan-4-yl) methyl ester, cis-	C_28_H_44_O_4_	444.6
2	11.45	2.436	Hexadecanoic acid, 1-(hydroxymethyl)-1,2-ethanediyl ester	C_35_H_68_O_5_	568.91
3	13.78	6.350	n-Hexadecanoic acid	C_16_H_32_O_2_	256.4
4	16.40	2.647	Octadecanoic acid	C_18_H_36_O_2_	284.48

**Table 6:** Chemical constituents present in the methanol extract of *N. laevis* stem bark from GC-MS analysis.

Peak number	Retention time (mins)	Area %	Compound name	Molecular formula	Molecular weight (g/Mol)
1	3.11	8.456	2-(3-Bromo-4-methoxy-4-methylcyclohexyl)propionitrile	C_11_H_18_BrNO	260.17
2	3.38	9.643	Maltol	C_6_H_6_O_3_	126.11
3	11.45	2.259	4-((1E)-3-hydroxy-1-propenyl)-2-methoxyphenol	C_10_H_12_O_3_	180.20
4	12.28	2.063	Benzoic acid, 4-hydroxy-3,5-dimethoxy-	C_9_H_10_O_5_	198.17

**Table 7:** Chemical constituents present in the methanol extract of *O. viride* leaves from GC-MS analysis.

Peak number	Retention time (mins)	Area %	Compound name	Molecular formular	Molecular weight (g/Mol)
1	5.95	6.571	Thymol	C_10_H_14_O	150.22
2	15.78	2.805	Phytol	C_20_H_40_O	128.17

**Table 8:** Behavioural and general physique observations of treatment and control groups under acute toxicity study.

Observation	Treatment group (2000 mg/kg)				Untreated
	MCL	SCSB	OVL	NLSB	Control
Body weight	Slight changes	Slight changes	Slight changes	Slight changes	No change
Diarrhoea	Absent	Absent	Absent	Absent	Absent
Tremor	Absent	Absent	Absent	Absent	Absent
Lethargy	None	None	None	None	None
Salivation	Absent	Absent	Absent	Absent	Absent
Convulsions	Absent	Absent	Absent	Absent	Absent
Eye colour	Normal	Normal	Normal	Normal	Normal
Food intake	Normal	Normal	Normal	Normal	Normal
Coma	None	None	None	None	None
Death	None	None	None	None	None

**Table 9:** Average and relative organ weights of rats administered with extracts.

Parameters	MCL	SCSB	OVL	NLSB	Ctrl
AOW					
Liver (g)	5.56 ± 0.80	4.84 ± 0.02	5.21 ± 1.21	5.07 ± 0.46	6.07 ± 0.32
Kidney (g)	0.49 ± 0.05	0.48 ± 0.02	0.52 ± 0.08	0.47 ± 0.02	0.55 ± 0.07

ROW					
BW (g)	164.25 ± 5.45	172.1 ± 1.80	194.05 ± 42.75	181.75 ± 1.25	206.3 ± 8.8
Liver (g)	3.38 ± 0.80	2.81 ± 0.02	2.68 ± 1.21	2.78 ± 0.46	2.9 ± 0.32
Kidney (g)	0.29 ± 0.05	0.27 ± 0.02	0.26 ± 0.08	0.25 ± 0.02	0.26 ± 0.07

Values are expressed as mean ± SEM, *n* = 5. MCL: *M. charantia* leaves; SCSB: *S. campanulate* stem bark; OVL: *O. viride* leaves; NLSB: *N. laevis* stem bark; CTR: control; SEM: standard error mean; AOW: average organ weight; ROW: relative organ weight; BW: body weight.

## Data Availability

The data obtained from this study have been included in this manuscript.

## Supplementary Materials

Supplementary Materials The chromatograms given as a result of gas chromatography-mass spectrometry (GC/MS) analysis of various plant extracts show active volatile compounds presented as prominent peaks at specific retention times and peak areas. The chromatogram of the methanol extract of *M. charantia* leaves (MCL) revealed five (5) prominent peaks with n-hexadecanoic acid (C_16_H_32_O_2_) having a larger peak area of 20.50% at a retention time (RT) of 14.128. The remaining peaks of MCL showed the presence of phytol (C_20_H_40_O), 3,7,11,15-tetramethyl-2-hexadecen-1-ol (C_20_H_40_O), thymol (C_10_H_14_O), and 1-chloromethyl-1-(2-propenyloxy)-1-silacyclohexane (C_7_H_13_Cl) at retention times of 16.071 (9.356%), 12.64 (2.681%), 6.152 (1.71%), and 3.20 (1.505%), respectively. *S. campanulata* stem bark (SCSB) shows four (4) prominent peaks: octadecenoic acid, (2-phenyl-1, 3-dioxolan-4-yl) methyl ester, *cis*- (C_28_H_44_O_4_), n-hexadecanoic acid (C_16_H_32_O_2_), octadecanoic acid (C_18_H_36_O_2_), and hexadecanoic acid, 1-(hydroxymethyl)-1, 2- (C_35_H_68_O_5_) at respective retention times and peak areas of 9.911 (6.444%), 13.78 (6.350%), 16.40 (2.647%), and 11.45 (2.436%). The chromatogram of *N. laevis* stem bark (NLSB) also showed four prominent peaks with maltol (C_6_H_6_O_3_) having the largest peak area of 9.643% at RT of 3.38 followed by 2-(3-bromo-4-methoxy-4-methylcyclohexyl)propionitrile (C_11_H_18_BrNO) at RT of 3.11 and peak area of 8.456%. The remaining peaks of NLSB with least peak areas of 2.259% and 2.063% represent 4-((1E)-3-hydroxy-1-propenyl)-2-methoxyphenol (C_10_H_12_O_3_) and benzoic acid, 4-hydroxy-3,5-dimethoxy- (C_9_H_10_O_5_) at retention times of 11.45 and 12.28, respectively. *O. viride* leaves (OVL) showed two (2) prominent peaks corresponding to thymol (C_10_H_14_O) and phytol (C_20_H_40_O) compounds occurring at respective RT of 5.95 and 15.78 with peak areas of 6.571% and 2.805%, respectively. The relative change in body weight of test rats administered with plant extract on the first day to the body weight of control rats (administered with water only) was observed and recorded weekly for 14 days, that is, the 7^th^ day (D7) and the 14^th^ day (D14). These observations showed a very minimal percentage of body weight changes among test rats to control rats as indicated by the graphs over the period of time.
