# Supplementary material for: In Vitro Cercaricidal Activity, Acute Toxicity, and GC/MS Analysis of Some Selected Ghanaian Medicinal Plants
Source: J Parasitol Res. 2023 Sep 15;2023:4589424. doi: 10.1155/2023/4589424 (PMC10516696; doi:10.1155/2023/4589424)
Supplement: Supplementary Materials — The chromatograms given as a result of gas chromatography-mass spectrometry (GC/MS) analysis of various plant extracts show active volatile compounds presented as prominent peaks at specific retention times and peak areas. The chromatogram of the methanol extract of M. charantia leaves (MCL) revealed five (5) prominent peaks with n-hexadecanoic acid (C16H32O2) having a larger peak area of 20.50% at a retention time (RT) of 14.128. The remaining peaks of MCL showed the presence of phytol (C20H40O), 3,7,11,15-tetramethyl-2-hexadecen-1-ol (C20H40O), thymol (C10H14O), and 1-chloromethyl-1-(2-propenyloxy)-1-silacyclohexane (C7H13Cl) at retention times of 16.071 (9.356%), 12.64 (2.681%), 6.152 (1.71%), and 3.20 (1.505%), respectively. S. campanulata stem bark (SCSB) shows four (4) prominent peaks: octadecenoic acid, (2-phenyl-1, 3-dioxolan-4-yl) methyl ester, cis- (C28H44O4), n-hexadecanoic acid (C16H32O2), octadecanoic acid (C18H36O2), and hexadecanoic acid, 1-(hydroxymethyl)-1, 2- (C35H68O5) at respective retention times and peak areas of 9.911 (6.444%), 13.78 (6.350%), 16.40 (2.647%), and 11.45 (2.436%). The chromatogram of N. laevis stem bark (NLSB) also showed four prominent peaks with maltol (C6H6O3) having the largest peak area of 9.643% at RT of 3.38 followed by 2-(3-bromo-4-methoxy-4-methylcyclohexyl)propionitrile (C11H18BrNO) at RT of 3.11 and peak area of 8.456%. The remaining peaks of NLSB with least peak areas of 2.259% and 2.063% represent 4-((1E)-3-hydroxy-1-propenyl)-2-methoxyphenol (C10H12O3) and benzoic acid, 4-hydroxy-3,5-dimethoxy- (C9H10O5) at retention times of 11.45 and 12.28, respectively. O. viride leaves (OVL) showed two (2) prominent peaks corresponding to thymol (C10H14O) and phytol (C20H40O) compounds occurring at respective RT of 5.95 and 15.78 with peak areas of 6.571% and 2.805%, respectively. The relative change in body weight of test rats administered with plant extract on the first day to the body weight of control rats (administered wit [file 4589424.f1.docx]

**GC-MS Chromatogram of extracts**

**5**

**4**

**3**

**1**

**2**

**Figure S1: GC-MS chromatogram of *M. charantia* leaf (MCL)**

**4**

**2**

**3**

**1**

**Figure S2: GC-MS chromatogram of *S. campanulata* stem bark (SCSB)**

**4**

**3**

**2**

**1**

**Figure S3: GC-MS chromatogram of *N. laevis* stem bark (NLSB)**

**2**

**1**

**Figure S4: GC-MS chromatogram of *O. viride* leave (OVL)**

**Effect of extract on weight of rats**

**Figure S5: Effects of extracts on weight of rats.** The percentage of total variation given the interaction between independent variable (days) and dependent variable (% body weight change) is **0.00037, 0.01498, 0.2183, 0.3890** at a *p-value* of **0.9957, 0.9719, 0.8967, 0.8605** for rats administered with ***M. charantia* (MC)**, ***S. campanulata* (SC),** ***O. viride* (O.V)** and ***N. laevis* (NL)** respectively versus control rats **(CTRL).** Therefore the weight change for all treatment rats relative to control rats (CTRL) are statistically not significant by a Two-way ANOVA analysis using GraphPad prism 8.0.1.
